# Radiomics with 3-dimensional magnetic resonance fingerprinting: influence of dictionary design on repeatability and reproducibility of radiomic features

**DOI:** 10.1007/s00330-022-08555-3

**Published:** 2022-03-18

**Authors:** Shohei Fujita, Akifumi Hagiwara, Koichiro Yasaka, Hiroyuki Akai, Akira Kunimatsu, Shigeru Kiryu, Issei Fukunaga, Shimpei Kato, Toshiaki Akashi, Koji Kamagata, Akihiko Wada, Osamu Abe, Shigeki Aoki

**Affiliations:** 1 Department of Radiology, Juntendo University School of Medicine, 1-2-1, Hongo, Bunkyo, Tokyo, 113-8421 Japan; 2 Department of Radiology, Graduate School of Medicine, The University of Tokyo, 7-3-1, Hongo, Bunkyo, Tokyo, 113-8654 Japan; 3 Department of Radiology, The Institute of Medical Science, The University of Tokyo, 4-6-1, Shiroganedai, Minato, Tokyo, 108-8639 Japan; 4 Department of Radiology, International University of Health and Welfare Narita Hospital, 852, Hatakeda, Narita, Chiba, 286-8520 Japan

**Keywords:** Brain, Magnetic resonance imaging, Neuroimaging, Repeatability, Reproducibility of results

## Abstract

**Objectives:**

We aimed to investigate the influence of magnetic resonance fingerprinting (MRF) dictionary design on radiomic features using in vivo human brain scans.

**Methods:**

Scan-rescans of three-dimensional MRF and conventional T1-weighted imaging were performed on 21 healthy volunteers (9 males and 12 females; mean age, 41.3 ± 14.6 years; age range, 22–72 years). Five patients with multiple sclerosis (3 males and 2 females; mean age, 41.2 ± 7.3 years; age range, 32–53 years) were also included. MRF data were reconstructed using various dictionaries with different step sizes. First- and second-order radiomic features were extracted from each dataset. Intra-dictionary repeatability and inter-dictionary reproducibility were evaluated using intraclass correlation coefficients (ICCs). Features with ICCs > 0.90 were considered acceptable. Relative changes were calculated to assess inter-dictionary biases.

**Results:**

The overall scan-rescan ICCs of MRF-based radiomics ranged from 0.86 to 0.95, depending on dictionary step size. No significant differences were observed in the overall scan-rescan repeatability of MRF-based radiomic features and conventional T1-weighted imaging (*p* = 1.00). Intra-dictionary repeatability was insensitive to dictionary step size differences. MRF-based radiomic features varied among dictionaries (overall ICC for inter-dictionary reproducibility, 0.62–0.99), especially when step sizes were large. First-order and gray level co-occurrence matrix features were the most reproducible feature classes among different step size dictionaries. T1 map-derived radiomic features provided higher repeatability and reproducibility among dictionaries than those obtained with T2 maps.

**Conclusion:**

MRF-based radiomic features are highly repeatable in various dictionary step sizes. Caution is warranted when performing MRF-based radiomics using datasets containing maps generated from different dictionaries.

**Key Points:**

• *MRF-based radiomic features are highly repeatable in various dictionary step sizes*.

• *Use of different MRF dictionaries may result in variable radiomic features, even when the same MRF acquisition data are used*.

• *Caution is needed when performing radiomic analysis using data reconstructed from different dictionaries*.

**Supplementary Information:**

The online version contains supplementary material available at 10.1007/s00330-022-08555-3.

## Introduction

Radiomics involves high-throughput computer extraction of potentially innumerable numbers of quantitative imaging metrics, or “radiomic features,” which are collectively used for prediction of disease diagnosis, treatment response, and prognosis [1–3]. In contrast to focal biopsy, which is an invasive procedure that only evaluates a small portion of tissue, radiomics allows the assessment of total pathology, including surrounding tissue and tracking of changes over time via repetitive non-invasive imaging. The implementation of radiomics into clinical practice has been challenging due to its sensitivity to various factors, such as image acquisition, imaging platform vendors, and feature extraction software, which affect the repeatability and reproducibility of radiomic features [4–8]. Integration of MRI-based radiomics into clinical workflow is challenging, particularly because the acquired signal intensity of MRI does not directly reflect the local physical properties and may differ substantially across imaging platforms [7, 9, 10]. Due to the qualitative nature of MRI, MRI-based radiomic features currently used in practice predominantly comprise morphometry (e.g., size, shape, and volume) of the structure, rather than histogram measurements or texture [5].

Magnetic resonance fingerprinting (MRF) is an image generation framework that can be employed to acquire quantitative maps of multiple tissue properties simultaneously [11]. In MRF, repetition times and flip angles are concurrently varied in a pseudorandom fashion to create through-time signals that characterize the various relaxation processes unique to each tissue type. These through-time signals are pattern-matched to separately simulated dictionary entries to restore measurable tissue properties. While the signal intensity of conventional MR images (such as T1- and T2-weighted images) depends on manifold acquisition parameters and MR scanner variations, MRF can generate highly repeatable and reproducible quantitative maps that have absolute scales [12–14]. Indeed, MRF is projected to emerge as the key technology for reproducible radiomic analyses and has been adopted for various sites, including the heart [15], breast [16, 17], prostate [18], liver [19], and brain [20–22], with promising results. MRF-based radiomics has been reported to improve the differentiation of common adult brain tumors by enabling the characterization of tumor heterogeneity and facilitating the prediction of outcomes in patients with glioblastoma [23].

Pattern-matching and dictionary design are active research areas due to the dependence of reconstructed MRF maps and reconstruction time on these processes [24]. Currently, there is substantial heterogeneity in dictionaries used for MRF, as various dictionaries with different step sizes are employed at different institutions [11, 13, 25–28]. To fully harness the quantitative maps generated by MRF across scanners and sites that are highly repeatable and reproducible, an analysis of maps reconstructed with different dictionaries is warranted.

Despite the potential of MRF-based radiomics, the influence of dictionary design on MRF-based radiomic features has not been investigated extensively. Herein, we investigated the influence of dictionary step size on the repeatability of MRF radiomic features and evaluated the stability of each feature using dictionaries with different step sizes.

## Materials and methods

### MRF acquisition and dictionary-matching

This study was conducted in compliance with the Image Biomarker Standardization Initiative guidelines [5, 29–31]. The methodology used for radiomics analysis is reported accordingly. The study was approved by the local institutional review board. Written informed consent was obtained from all participants prior to the scan. Twenty-one participants (9 males and 12 females; mean age, 41.3 ± 14.6 years; age range, 22–72 years) with no history of neurological or psychological disorders were enrolled. Five patients with multiple sclerosis (3 males and 2 females; mean age, 41.2 ± 7.3 years; age range, 32–53 years, median Expanded Disability Status Scale, 1 [range, 0–7.5]; mean disease duration, 11.2 ± 4.4 years [range, 4–18 years]) were also included. Only inter-dictionary reproducibility was evaluated in patients because undergoing scan-rescan was not feasible. All subjects underwent non-contrast-enhanced brain scans using a 3-T scanner (Discovery 750 w, GE Healthcare) with a standard 32-channel head coil. No motion correction techniques were applied.

Scan-rescan was performed using an identical protocol consisting of whole-brain 3D MRF and conventional 3D fast spoiled gradient echo (FSPGR) imaging. After the first imaging set (consisting of an MRF scan and FSPGR scan) was acquired, participants exited the room and were repositioned before the rescan. The scanner was calibrated before each set of scans. The 3D MRF sequence was based on steady-state free precession with spiral projection k-space trajectory [14, 32]. The acquisition parameters of MRF were as follows: field of view, 200 × 200 × 200 mm; matrix size, 200 × 200 × 200; spatial resolution, 1.0 × 1.0 × 1.0 mm; and acquisition time, 9 min 51 s. The acquisition parameters of conventional 3D T1-weighted structural images were as follows: acquisition orientation, sagittal acquisition; TR/TE/inversion time, 7.7/3.1/400 ms; field of view, 256 × 256 mm; matrix size, 256 × 256; section thickness, 1.0 mm; spatial resolution, 1.0 × 1.0 × 1.0 mm; flip angle, 11°; receiver bandwidth, 244.1 Hz/pixel; number of excitations, 1; and acquisition time, 5 min 45 s. The spatial resolution of 3D MRF and 3D FSPGR was matched to gapless isotropic 1.0 mm.

Reconstructions and dictionary-matching were performed using an in-house program in MATLAB (R2019a, MathWorks). Dictionaries were generated using the extended phase graph formalism [11]. To generate a set of dictionaries with different step sizes, various step sizes were prepared as shown in Table 1. The dictionary T1 range and T2 range were kept the same across dictionaries, ranging from 10 to 3000 ms and 10 to 1000 ms, respectively. In all dictionaries, we used smaller step sizes for T2 than for T1 because T2 is smaller than T1 in biological tissues. MRF T1 and T2 maps were obtained using a maximum inner product search [11].

**Table 1:** Step sizes and total number of entries for each dictionary. All dictionaries had the same range of 10 to 3000 ms for T1 and 10 to 1000 ms for T2

Dictionary name	Highly dense	Dense	Moderate	Sparse	Highly sparse
T1 step size (ms)	10	20	40	80	100
T2 step size (ms)	2	4	8	16	20
No. of entries	148,005	36,803	9102	2257	1421

### Data post-processing and radiomic feature extraction

**Fig. 1:**
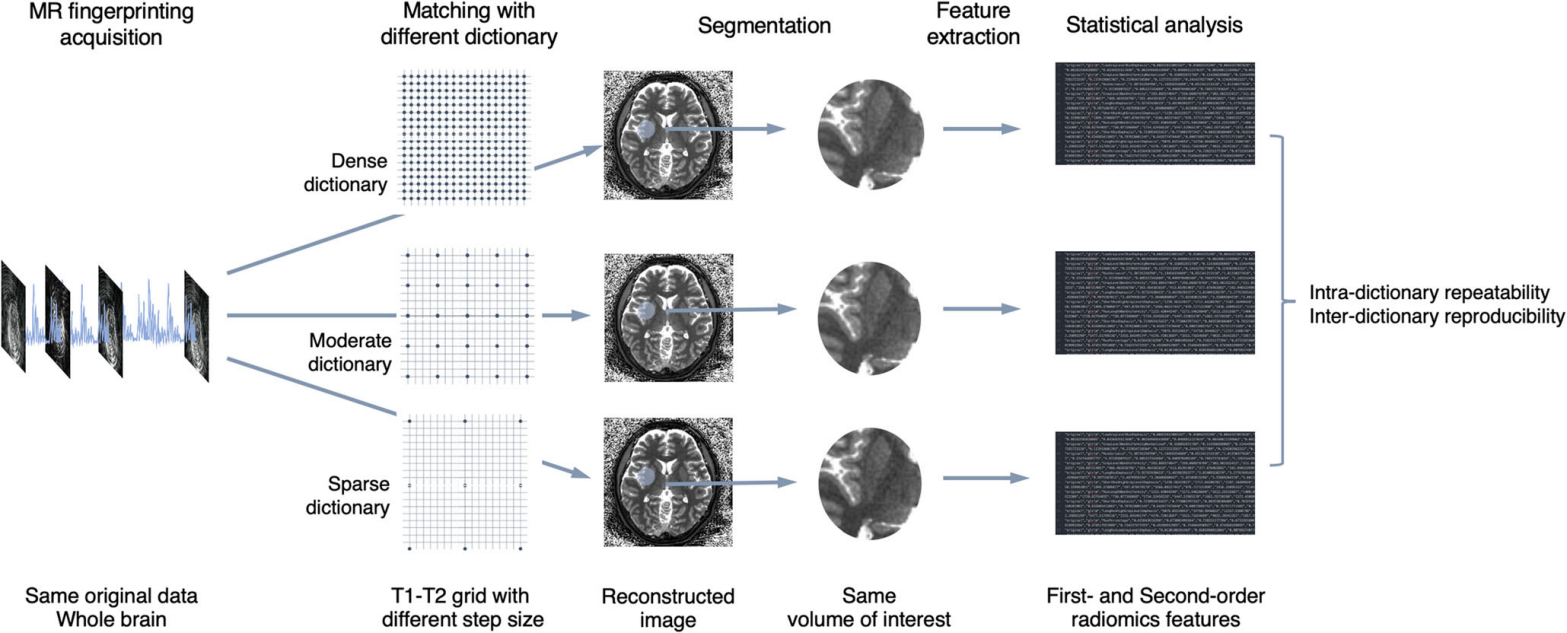
Schematic overview of the evaluation process of the effect of magnetic resonance fingerprinting (MRF) dictionary design on radiomic features. Various dictionaries with different step sizes were applied to the same MRF acquisition data to reconstruct quantitative maps. Identical volumes of interest were applied to each of these maps to extract radiomic features, which were then used to evaluate intra-dictionary repeatability and inter-dictionary reproducibility for each radiomic feature

**Fig. 2:**
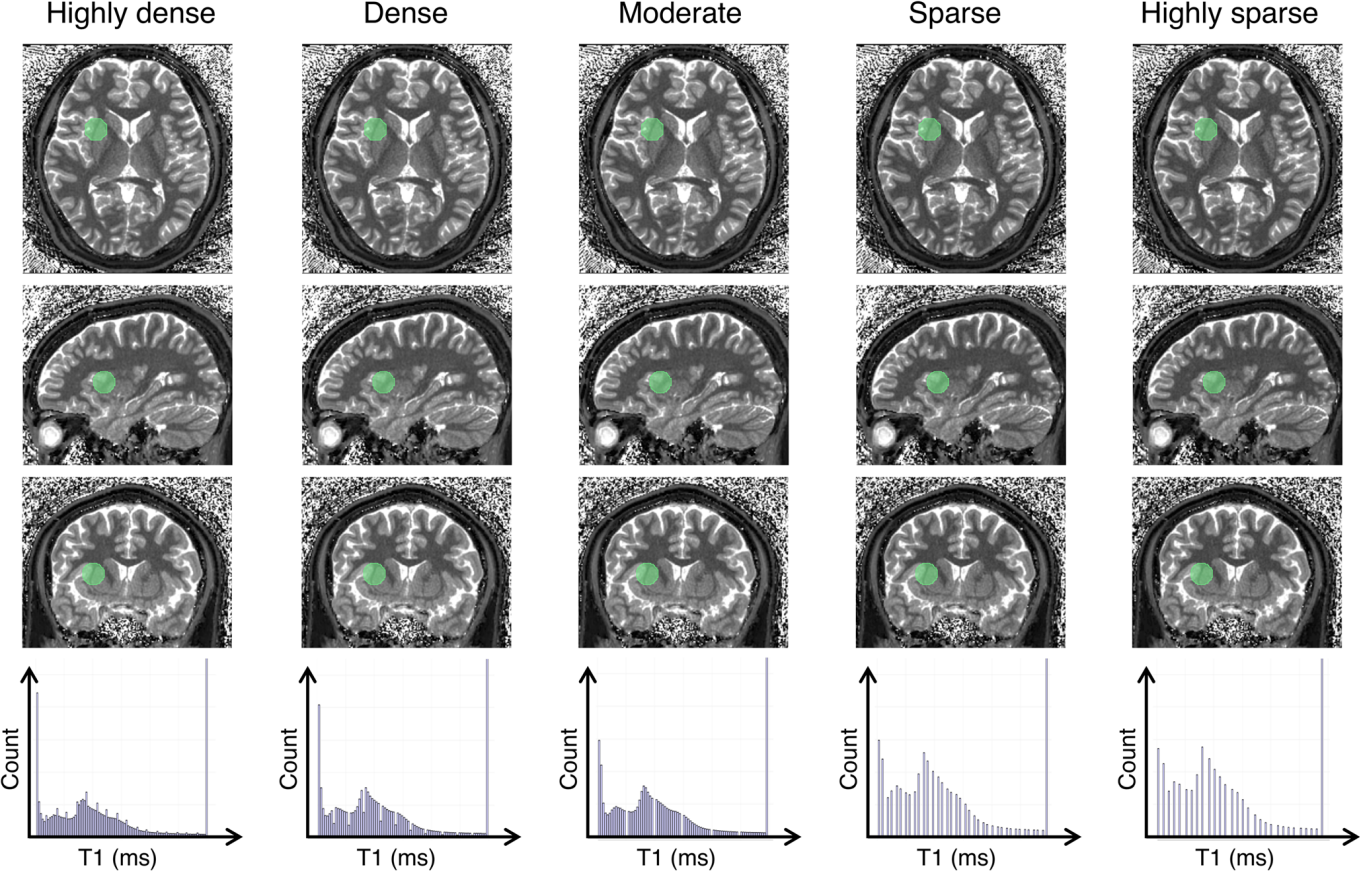
Representative magnetic resonance fingerprinting (MRF) maps and a histogram of each dictionary with different step sizes. Representative T1 maps generated from different dictionaries in axial, coronal, and sagittal views (upper 3 rows). Representative spherical volume of interest with a diameter of 20 mm is indicated by transparent green color. Since datasets for each subject are inherently aligned in MRF, each volume of interest was copied and pasted across all datasets. Note that the images obtained from different dictionaries are challenging to distinguish visually. Histogram of T1 values for each dictionary (bottom row). Note that the sparser dictionary has coarser discretization values

An overview of data post-processing is illustrated in Fig. 1. Skull-stripping was performed using the *bet* function implemented in FMRIB Software Library (version 6.0.4; FMRIB Analysis Group). Spherical volumes of interest (VOIs) with a diameter of 20 mm were randomly and manually placed inside the skull on the first FSPGR image (Fig. 2) using the “Segment Editor” module in 3D Slicer (version 4.10.2, https://www.slicer.org/) [33]. Additionally, ellipsoid (axes, 20 × 12 mm) and cubic (12 mm each side) VOIs were also prepared to investigate the effect of dictionary design on the reproducibility of radiomic features under various VOI shapes. We did not align the MRF images and rescan FSPGR to initial FSPGR images to avoid intensity interpolation, which may have altered the signal differences originally contained within the data. Instead, VOIs set on initial FSPGR images were rigidly translated to the rescan FSPGR space, first-scan MRF space, and rescan MRF space using the *flirt* function implemented in FMRIB Software Library. Since all MRF datasets for each subject were inherently aligned (highly dense, dense, moderate, sparse, and highly sparse datasets were reconstructed from the same acquisition data), each VOI was copied and pasted across MRF datasets of the same subject. In total, 12,600 VOIs from healthy volunteers (21 subjects, 300 VOIs per subject, scan-rescan dataset) and 1250 VOIs from patients with multiple sclerosis (5 patients, 250 VOIs per subject, scan dataset) were used in subsequent analyses.

PyRadiomics (version 3.0) [34], an open-source radiomics software package that is compliant with the IBSI benchmarks [31], was used to extract first- and second-order features from the VOIs for each dataset as defined in default by PyRadiomics. Briefly, first-order features describe the distribution of voxel intensities within a VOI, whereas second-order features express combinations of voxel intensities of neighboring pixels distributed within a VOI. Second-order features included symmetrical gray level co-occurrence matrix (GLCM), gray level run length matrix (GLRLM), gray level size zone matrix (GLSZM), and neighboring gray tone difference matrix (NGTDM). These features complied with feature definitions as described by the IBSI, which are available in a separate reference manual by Zwanenburg et al [7]. Bin counts of 64 were used because gray levels of 32 to 64 typically enable radiomic analysis without losing important features in medical imaging [3, 10, 23, 35]. No image resampling, image intensity normalization, or image filtering was performed.

### Statistical analysis

To evaluate intra-dictionary repeatability, two-way mixed-effects models of the intraclass correlation coefficients (ICCs, unit: single rater/measurement, type: absolute agreement) and their 95% confidence intervals (CIs) were calculated for each feature value extracted from scans and rescans [36]. To evaluate reproducibility, two-way random-effects models of the ICCs (unit: single rater/measurement, type: agreement, consistency) and their 95% CIs were calculated against the reference value for each feature as a measure of the agreement between the highly dense dictionary and others. This analysis was performed to evaluate the stability of features against differences in dictionary step size. The radiomic features obtained from the densest dictionary were used as reference values, as they were considered to contain the greatest amount of information. Only the first scan was used to calculate reproducibility because only one scan is available (rescan is not performed) in clinical settings. Negative ICC estimates were truncated at zero. To evaluate the effects of different step sizes on radiomic features, percent relative changes were calculated with respect to corresponding references. ICCs exceeding 0.90 were categorized as high performance and acceptable, in accordance with thresholds reported in the literature [37–39].

All statistical analyses were performed using R statistical software (version 3.5.1; R Foundation for Statistical Computing) with packages “psych” (version 1.8.10) and “tidyverse” (version 1.2.1). The Wilcoxon rank sum test was used to compare individual radiomic features across dictionaries. Results were considered significant if *p* values were below the significance threshold (0.05 divided by the total number of combinations of the dictionaries) after applying the Bonferroni method for multiple-comparison correction. The significance threshold for adjusted *p* values was set at 0.05.

## Results

### Scan-rescan repeatability of MRF-based radiomic features using dictionaries with different step sizes

**Fig. 3:**
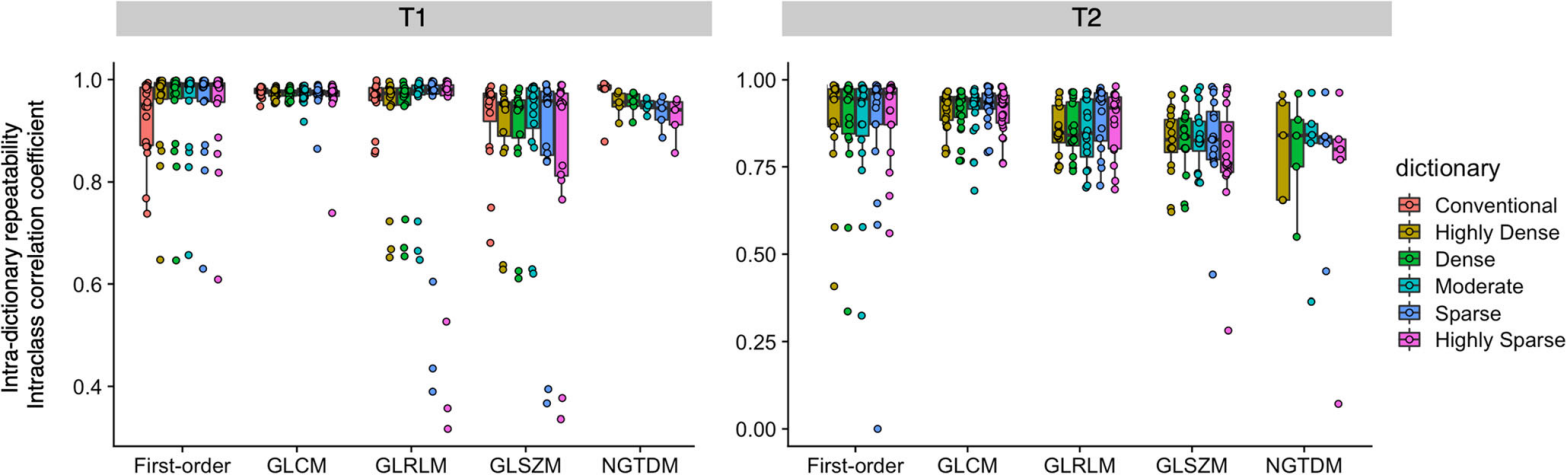
Scan-rescan within-dictionary repeatability of magnetic resonance fingerprinting (MRF)–derived radiomic features across dictionaries with different step sizes. Spherical volume of interests was used. Intraclass correlation coefficients computed for the entire study population are depicted in boxplots. Boxes indicate the interquartile range (25–75%), and circles indicate radiomic features. GLCM, gray level co-occurrence matrix; GLRLM, gray level run length matrix; GLSZM, gray level size zone matrix; NGTDM, neighboring gray tone difference matrix

**Fig. 4:**
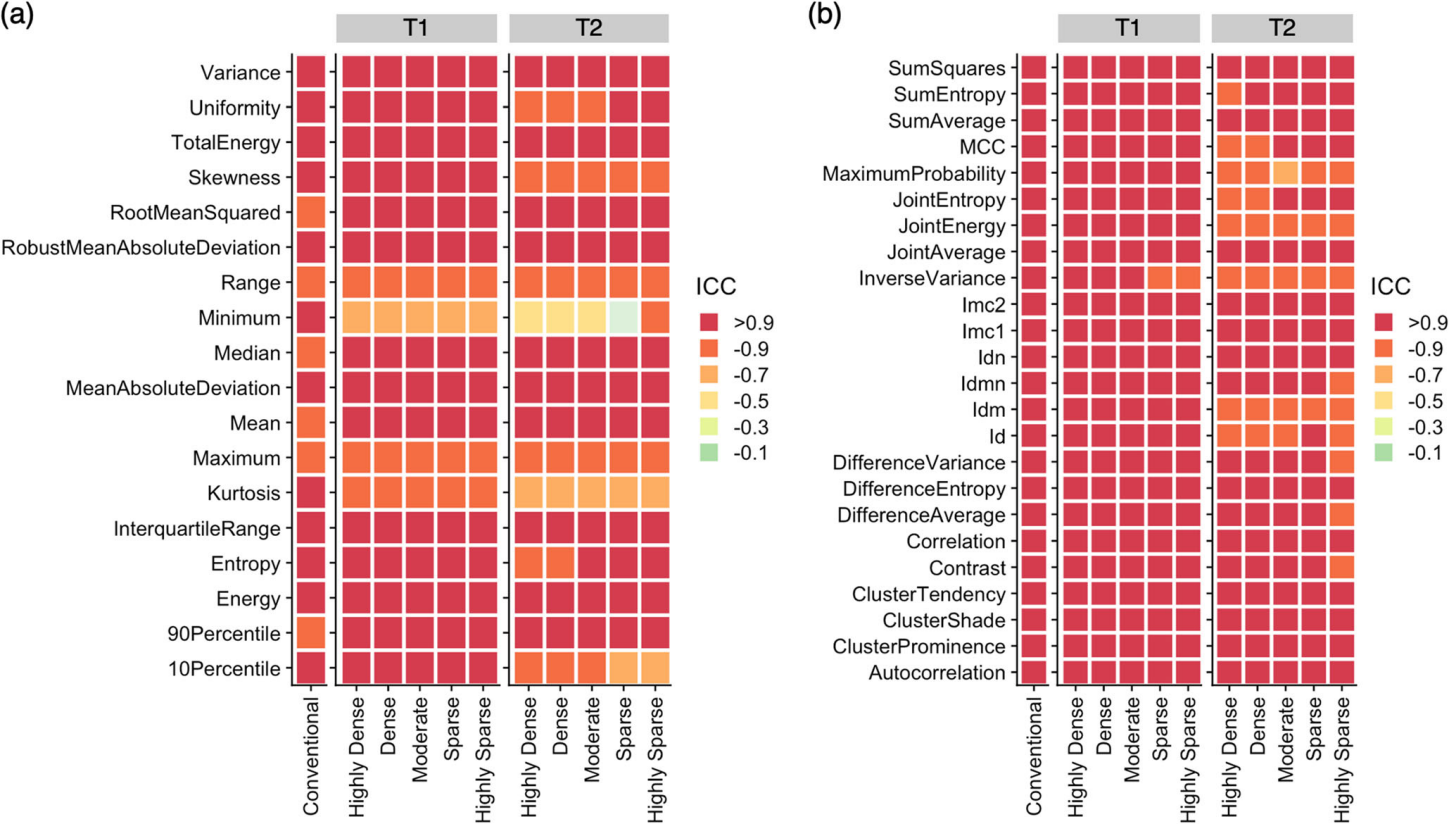
Intra-dictionary repeatability of magnetic resonance fingerprinting (MRF)–derived radiomic features across conventional imaging and MRF with dictionaries of different step sizes. (**a**) First-order features and (**b**) symmetrical gray level co-occurrence matrix are shown. Spherical volume of interests was used. Conventional refers to conventional 3D T1-weighted imaging. ICC, intraclass correlation coefficient; Id, inverse difference; Idn, inverse difference normalized; Idm, inverse difference moment; Idmn, inverse difference moment normalized; Imc, informational measure of correlation; MCC, maximal correlation coefficient

Intra-dictionary scan-rescan ICCs of radiomic features derived from MRF using different step sizes and conventional imaging are presented in Fig. 3. Repeatability of individual radiomic features is presented in Fig. 4 (first-order and symmetric GLCM), and see Electronic Supplementary Material Figure 1 (GLRLM, GLSZM, and NGTDM). ICCs were computed based on the entire study population. Intra-dictionary repeatability was generally insensitive to dictionary step sizes. Overall scan-rescan ICCs for conventional 3D T1-weighted imaging and highly dense, dense, moderate, sparse, and highly sparse dictionaries for MRF T1-derived radiomic features were 0.95 ± 0.06, 0.94 ± 0.09, 0.94 ± 0.09, 0.94 ± 0.9, 0.93 ± 0.14, and 0.92 ± 0.16, respectively (mean ± standard deviation). No significant differences were observed among different MRF dictionaries (*p* = 1.0–1.0). T1 map-derived GLCM features tended to have poorer repeatability in sparser dictionaries. Among dictionaries, the highly dense dictionary provided the highest number of highly repeatable (i.e., ICC > 0.90) radiomic features (*n* = 68/79, 86%), which was noninferior to conventional imaging (*n* = 65/79, 83%) (*p* = 0.82). T2 maps generally exhibited poorer intra-dictionary repeatability compared to T1 maps. Overall scan-rescan ICCs for highly dense, dense, moderate, sparse, and highly sparse dictionaries for T2-derived radiomic features were 0.87 ± 0.11, 0.87 ± 0.11, 0.86 ± 0.12, 0.87 ± 0.15, and 0.86 ± 0.15, respectively (mean ± standard deviation). No significant differences were noted in the repeatability of radiomic features derived from T2 maps among different MRF dictionaries (*p* = 1.0–1.0). The percentage of highly repeatable T2 map-derived radiomic features provided by the highly dense dictionary was 46% (*n* = 36/79).

The percentages of high repeatability (ICC > 0.90) of each feature class among T1 and T2 maps among dictionaries were 69% (*n* = 124/180), 85% (*n* = 205/240), 63% (*n* = 100/160), 44% (*n* = 71/160), and 58% (*n* = 29/50) for first-order, GLCM, GLRLM, GLSZM, and NGTDM features, respectively. Poor repeatability was observed in the range, minimum, and maximum for both T1 and T2 maps, and kurtosis of T2 maps. Features related to lower gray level values (e.g., run low gray level emphasis) exhibited poorer repeatability with increased dictionary step size using spherical VOIs.

The results using ellipsoid and cubic VOIs were similar to those obtained with spherical VOIs (Electronic Supplementary Material Figure 2), showing high intra-dictionary repeatability and general insensitivity to dictionary step sizes.

### Inter-dictionary reproducibility of MRF-based radiomic features

**Fig. 5:**
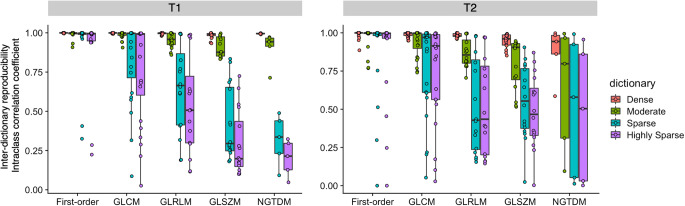
Reproducibility of radiomic features calculated with magnetic resonance fingerprinting (MRF) using dictionaries with different step sizes. Intraclass correlation coefficients were computed using radiomic features obtained from the highly dense dictionary as a reference for the entire study population. Spherical volume of interests was used. Boxes indicate the interquartile range (25–75%), and circles indicate radiomic features. GLCM, gray level co-occurrence matrix; GLRLM, gray level run length matrix; GLSZM, gray level size zone matrix; NGTDM, neighboring gray tone difference matrix

**Fig. 6:**
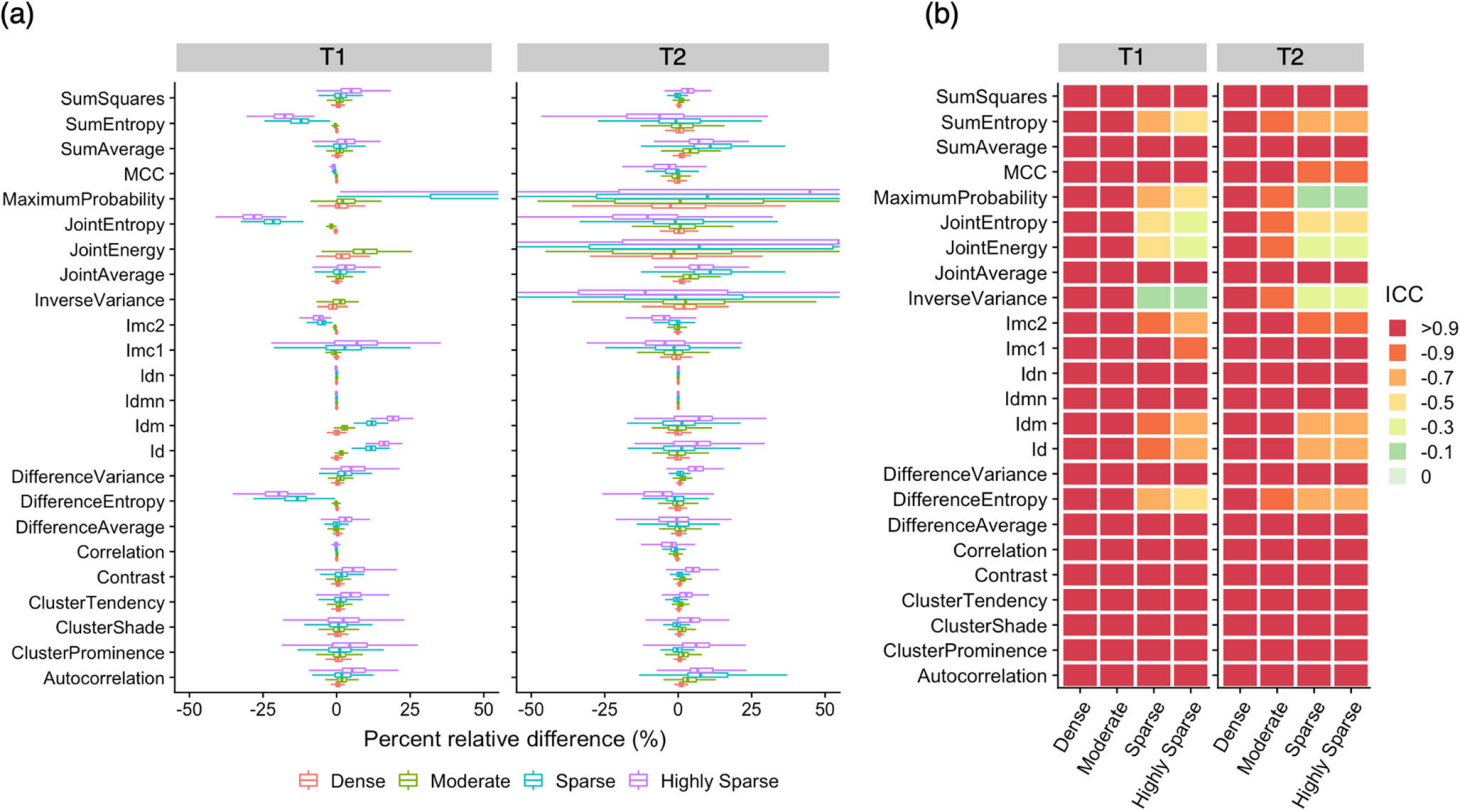
Effect of magnetic resonance fingerprinting (MRF) dictionary step size on symmetrical gray level co-occurrence matrix. (**a**) Inter-dictionary percent relative change of radiomic features. (**b**) Intraclass correlation coefficients (ICCs) for each feature (rows) extracted with different dictionary step sizes (columns). Radiomic features obtained from the highly dense dictionary were used as references based on the assumption that the highly dense dictionary contained the greatest amount of information. Spherical volume of interests was used. Id, inverse difference; Idn, inverse difference normalized; Idm, inverse difference moment; Idmn, inverse difference moment normalized; Imc, informational measure of correlation; MCC, maximal correlation coefficient

The inter-dictionary reproducibility of radiomic features calculated with MRF using different step size dictionaries is presented in Fig. 5. The percent relative differences and ICCs of first-order and symmetric GLCM features are presented in Electronic Supplementary Material Figure 3 and Figure 6, respectively (see Electronic Supplementary Material Figures 4–6, which illustrate the inter-dictionary reproducibility of GLRLM, GLSZM, and NGTDM features). ICCs were computed based on the entire study population. Across all feature classes, features calculated using the dense dictionary generally exhibited greater agreement with those calculated with the reference (i.e., highly dense dictionary). Radiomic features calculated using the dense dictionary exhibited high reproducibility (ICC > 0.90) with those calculated using the highly dense dictionary (T1, *n* = 79/79, 100%; T2, *n* = 73/79, 92%). Reproducibility decreased with an increase in dictionary step size. The reproducibility ICCs (mean ± standard deviation) for dense, moderate, sparse, and highly sparse dictionaries in T1 maps were 0.99 ± 0.01, 0.96 ± 0.06, 0.70 ± 0.31, and 0.62 ± 0.36, respectively. For T2 maps, the reproducibility ICCs (mean ± standard deviation) for dense, moderate, sparse, and highly sparse dictionaries were 0.97 ± 0.06, 0.88 ± 0.16, 0.69 ± 0.33, and 0.66 ± 0.34, respectively. Significant differences were observed in all dictionary combinations for both T1 and T2 maps (*p* < 0.001), except for highly sparse and sparse dictionaries (*p* = 0.22 and 1.00 for T1 and T2, respectively).

First-order features were generally more reproducible than second-order features. Reproducibility of GLRLM, GLSZM, and NGTDM features was sensitive to an increase in dictionary step size. Among first-order features, entropy and uniformity were the most sensitive to changes in dictionary step size and exhibited a relative difference of > 25% compared to reference values (Electronic Supplementary Material Figures 3). Results for sparser dictionaries were generally more non-reproducible for second-order features than for first-order features.

Radiomic features calculated using T1 maps exhibited smaller percent relative differences among dictionaries than those calculated using T2 maps (Figs. 5 and 6; see Electronic Supplementary Material Figures 3–6). Overall, T2-derived radiomic features exhibited greater variability compared to T1-derived radiomic features. In contrast, T1-derived radiomic features exhibited less variability but larger bias (i.e., deviation from the highly dense dictionary, indicated by the magnitude of percent relative differences). This tendency was most evident in NGTDM (see Electronic Supplementary Material Figures 4–6).

The inter-dictionary reproducibility using ellipsoid and cubic VOIs was similar to those obtained with spherical VOIs (Electronic Supplementary Material Figure 2). Across all feature classes, features calculated using the dense dictionary generally exhibited greater agreement with those calculated with the reference (i.e., highly dense dictionary), and reproducibility decreased with an increased dictionary step size.

The inter-dictionary reproducibility of radiomic features in patients with multiple sclerosis is summarized in Electronic Supplementary Material Figure 7. The results were similar to those for healthy subjects. Across all feature classes, features calculated using the dense dictionary generally exhibited greater agreement with those calculated with the reference (i.e., highly dense dictionary). The reproducibility ICCs (mean ± standard deviation) for dense, moderate, sparse, and highly sparse dictionaries in T1 maps were 0.99 ± 0.02, 0.96 ± 0.07, 0.71 ± 0.31, and 0.64 ± 0.35, respectively. For T2 maps, the reproducibility ICCs for dense, moderate, sparse, and highly sparse dictionaries were 0.98 ± 0.05, 0.89 ± 0.16, 0.73 ± 0.30, and 0.68 ± 0.32, respectively.

## Discussion

Due to its highly repeatable and reproducible quantitative maps, MRF has emerged as a key technology for reliable radiomic analyses. Maps derived using MRF may be used in multi-center radiomic studies by pooling the data without the need for normalization or harmonization [40] that could scale away important information originally contained within the images. Nevertheless, the influence of dictionary design, a crucial component of MRF, on radiomic features has not been comprehensively investigated. This study evaluated the influence of dictionary design on intra-dictionary repeatability and inter-dictionary reproducibility of extracted radiomic features. Our results demonstrated that (i) repeatability of MRF-based radiomic features is unaffected by the dictionary step size, and (ii) the use of different MRF dictionaries may result in variable radiomic features, even when the same MRF acquisition data are used, and (iii) these results were consistent even when using different VOI shapes for volunteers and patients. Therefore, maps obtained with different dictionaries may produce erroneous radiomic analyses when used simultaneously.

The repeatability of MRF-derived radiomic features using the highly dense dictionary was comparable to conventional imaging in our study (89%, 87%, and 82% for MRF T1 maps, MRF T2 maps, and conventional imaging, respectively). The repeatability observed in this study was generally higher than values reported in the literature based on conventional MRI. Baessler et al [9] reported that the percentage of robust scan-rescan features obtained from conventional T1-weighted images was 54% (*n* = 25/45), whereas MRF using the highly dense dictionary generated repeatability of 89%. Although a direct comparison is challenging since we used a different radiomics platform and Baessler et al used vegetables and fruits rather than the human brain for imaging, our results demonstrate that MRF with a highly dense dictionary provides repeatable radiomic features.

Although our results indicate that merging of MRF-derived radiomic features calculated using different step size dictionaries should be avoided, several features exhibited high reproducibility across different dictionaries. First-order features and GLCM were generally more reproducible compared to other second-order features, in accordance with previous literatures [30, 41]. We identified several features with high reproducibility (ICC > 0.90) across dictionaries. This indicates the possibility of merging MRF-derived radiomic features calculated using different step size dictionaries. For example, Badve et al reported that mean T1 and T2 values and T2 skewness exhibited significant differences between solid tumor regions in glioblastoma and low-grade gliomas [22]. These features were highly reproducible across different dictionaries (ICC > 0.90 for both T1 and T2), highlighting the feasibility of pooling data from different dictionaries for this purpose. MRF-based radiomic analyses of adult brain tumors by Dastmalchian et al. revealed that inverse differences were normalized and homogeneity (equivalent of inverse difference) values of peritumoral white matter provided the best discrimination of low-grade gliomas, glioblastomas, and metastases [23]. Our results suggest that inverse differences have high reproducibility among highly dense, dense, and moderate dictionaries, and normalized inverse differences are highly reproducible (ICC > 0.90 among all dictionaries) and repeatable (ICC > 0.90, except for T2 of the highly sparse dictionary).

Several limitations of our study should be acknowledged. First, although a wide age range of healthy volunteers was included, the sample size of patients was small. Thus, we only evaluated the influence of dictionary design on radiomic features but not on downstream analysis in clinically relevant predictive models. Radiomic features are conveyed to machine-learning models for use in certain tasks, such as predicting diagnosis, treatment response, and prognosis. Performing MRF-based radiomics on real patient data to evaluate the impact of dictionary design on resulting predictive models is warranted in the future. This may enable accurate radiomic analysis of MRF data from different dictionaries used simultaneously. Second, we used a single sequence with fixed acquisition parameters, which may overlook the flexibility and robustness of the MRF framework. It would be interesting to investigate these effects on radiomic features in a future study. Third, we employed a single scanner study design and were thus unable to evaluate the inter-scanner reproducibility of MRF-based radiomic features in this study. Due to the high reproducibility of MRF T1 and T2 maps across scanners, the inter-scanner reproducibility of MRF-based radiomic features may achieve greater reproducibility than conventional qualitative imaging. This would be of substantial interest in clinical settings and warrants further investigation. Nevertheless, given the current paucity of investigations on repeatability and dictionary dependence of MRF-based radiomic features, our findings serve as a baseline and provide fundamental information which will facilitate clinical integration of MRF-based radiomics.

Our findings indicate that MRF-based radiomic features are highly repeatable across various dictionary step sizes. The repeatability of MRF-based radiomics is insensitive to dictionary step size. Based on our results, except for a small subset of radiomic features, we recommend against performing radiomic analysis using data reconstructed from different dictionaries.

## Supplementary Information

ESM 1 (DOCX 3768 kb)

## Abbreviations

CI: Confidence interval
FSPGR: Fast spoiled gradient echo
GLCM: Gray level co-occurrence matrix
GLRLM: Gray level run length matrix
GLSZM: Gray level size zone matrix
ICCs: Intraclass correlation coefficients
MRF: Magnetic resonance fingerprinting
NGTDM: Neighboring gray tone difference matrix
VOI: Volume of interest

## Declarations

### Guarantor

The scientific guarantor of this publication is Shigeki Aoki.

### Statistics and biometry

One of the authors has significant statistical expertise.

### Study subjects or cohorts overlap

Some study subjects or cohorts have been previously reported in Fujita et al *Hum Brain Mapp* 2021.

### Methodology

• prospective

• experimental

• performed at one institution
