# Supplementary material for: Radiomics with 3-dimensional magnetic resonance fingerprinting: influence of dictionary design on repeatability and reproducibility of radiomic features
Source: Eur Radiol. 2022 Mar 18;32(7):4791–800. doi: 10.1007/s00330-022-08555-3 (PMC9213334; doi:10.1007/s00330-022-08555-3)

**Electronic Supplementary Material**

**Electronic Supplementary Material Figure 1.** Intra-dictionary repeatability of magnetic resonance fingerprinting (MRF)-derived radiomic features across conventional imaging and MRF with dictionaries of different step sizes. (a) Gray level run length matrix, (b) gray level size zone matrix, and (c) neighboring gray tone difference matrix are presented. ICC, intraclass correlation coefficient.

**
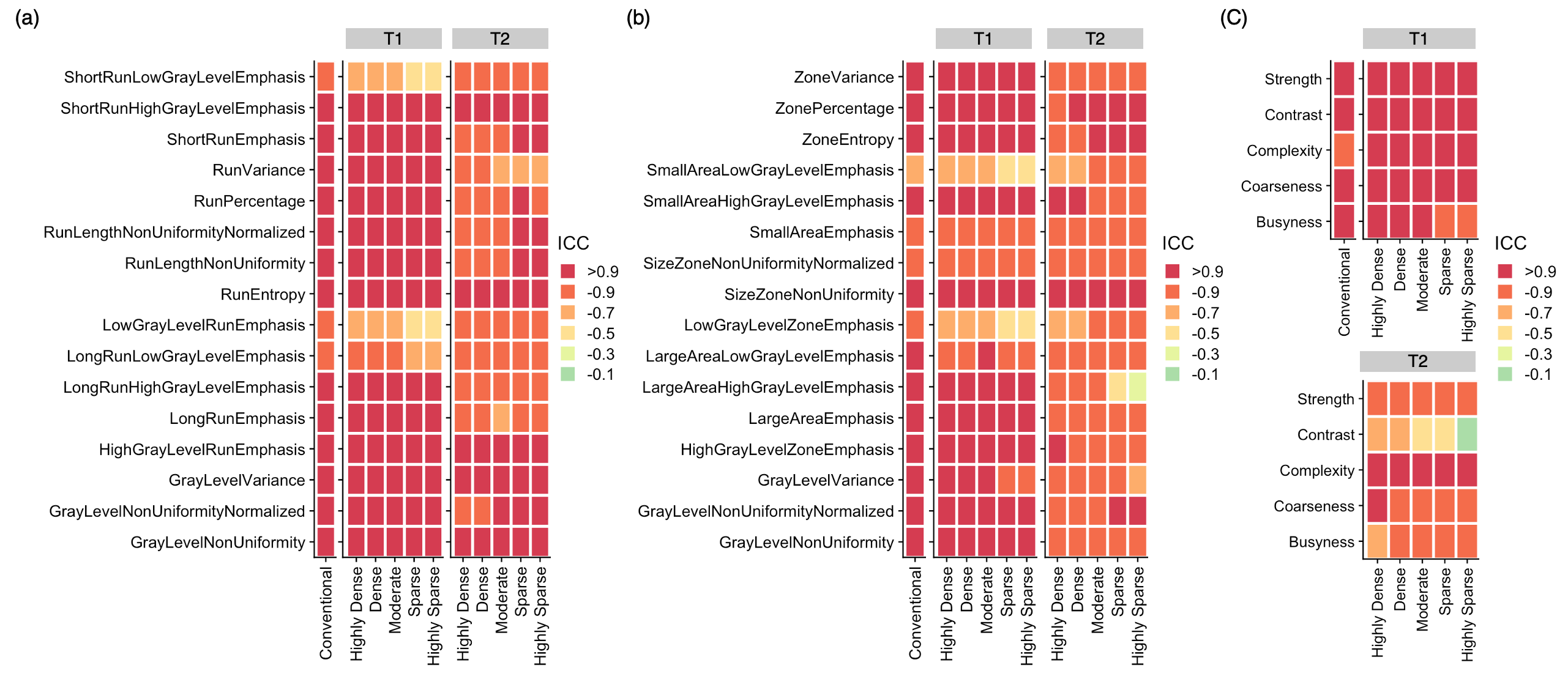
**

**Electronic Supplementary Material Figure 2.** Repeatability and reproducibility of radiomic features calculated with magnetic resonance fingerprinting (MRF) with (a) cube and (b) ellipsoid volume of interests. Scan-rescan intraclass correlation coefficients indicating within-dictionary repeatability were computed for the entire study population. Inter-dictionary reproducibility of radiomic features was evaluated with intraclass correlation coefficients computed for the entire study population using features obtained from the highly dense dictionary as a reference. Boxes indicate the interquartile range (25–75%), and circles indicate radiomic features.

**
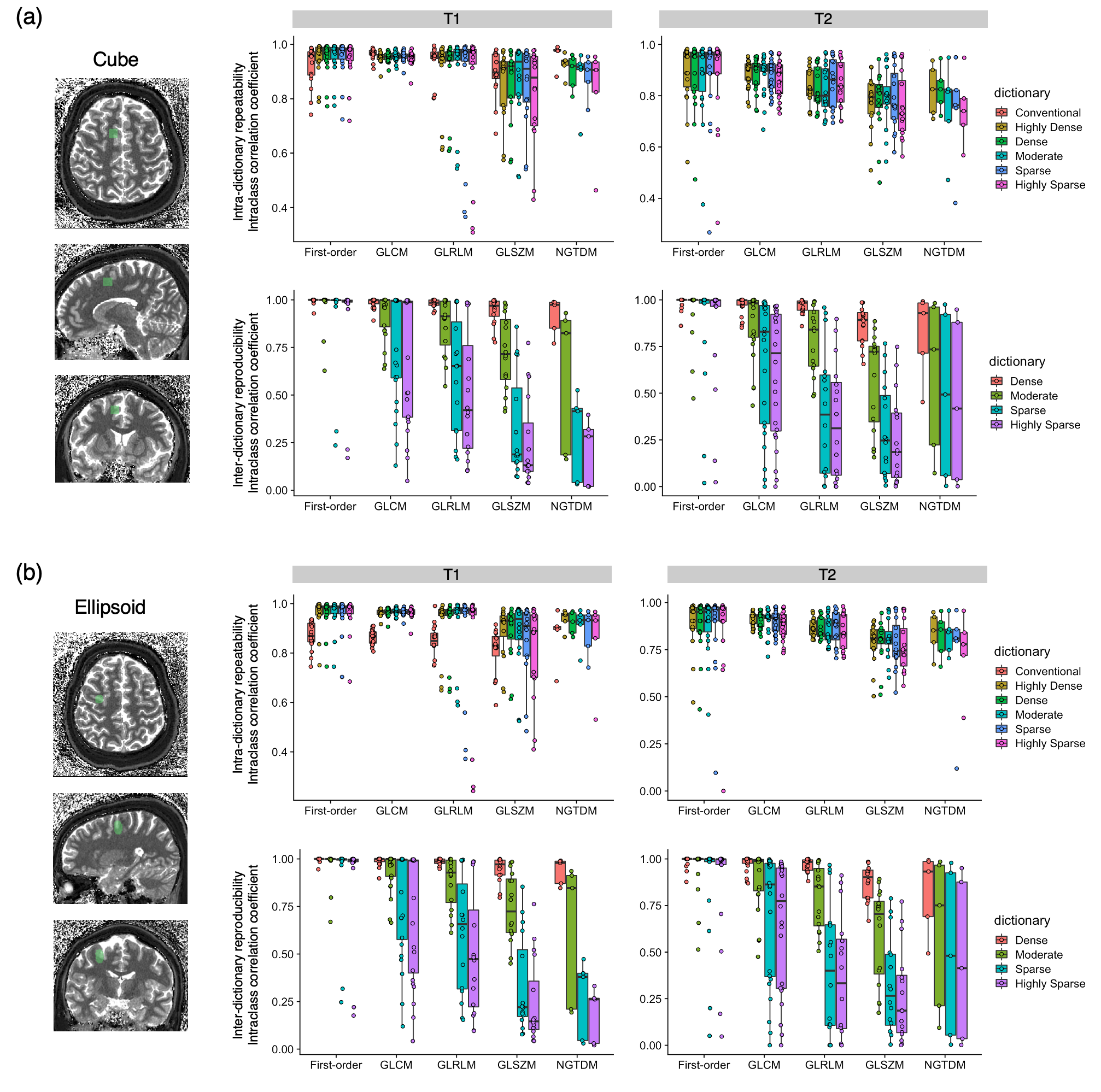
**

**Electronic Supplementary Material Figure 3.** Effect of magnetic resonance fingerprinting (MRF) dictionary step size on first-order features. (a) Inter-dictionary percent relative change of radiomic features. (b) Intraclass correlation coefficients (ICCs) of first-order features (rows) extracted with various step sizes (columns). Entropy exhibited high relative differences and low ICCs. Radiomic features obtained from the highly dense dictionary were used as references based on the assumption that the highly dense dictionary contained the greatest amount of information.

**
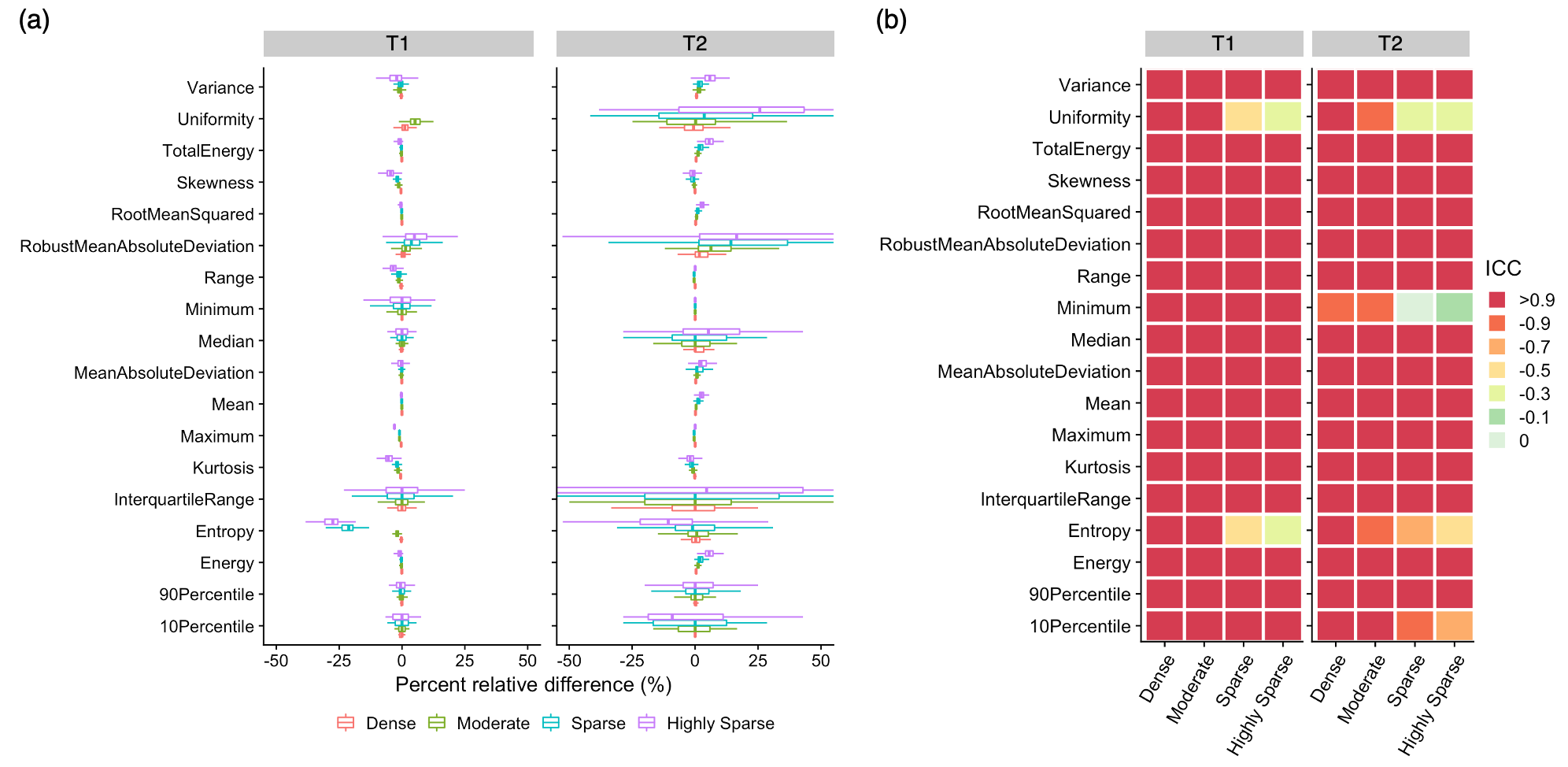
**

**Electronic Supplementary Material Figure 4.** Effect of magnetic resonance fingerprinting dictionary step size on gray level run length matrix. (a) Inter-dictionary percent relative changes of features. (b) Intraclass correlation coefficients (ICCs) for each feature (rows) extracted using different dictionary step sizes (columns).


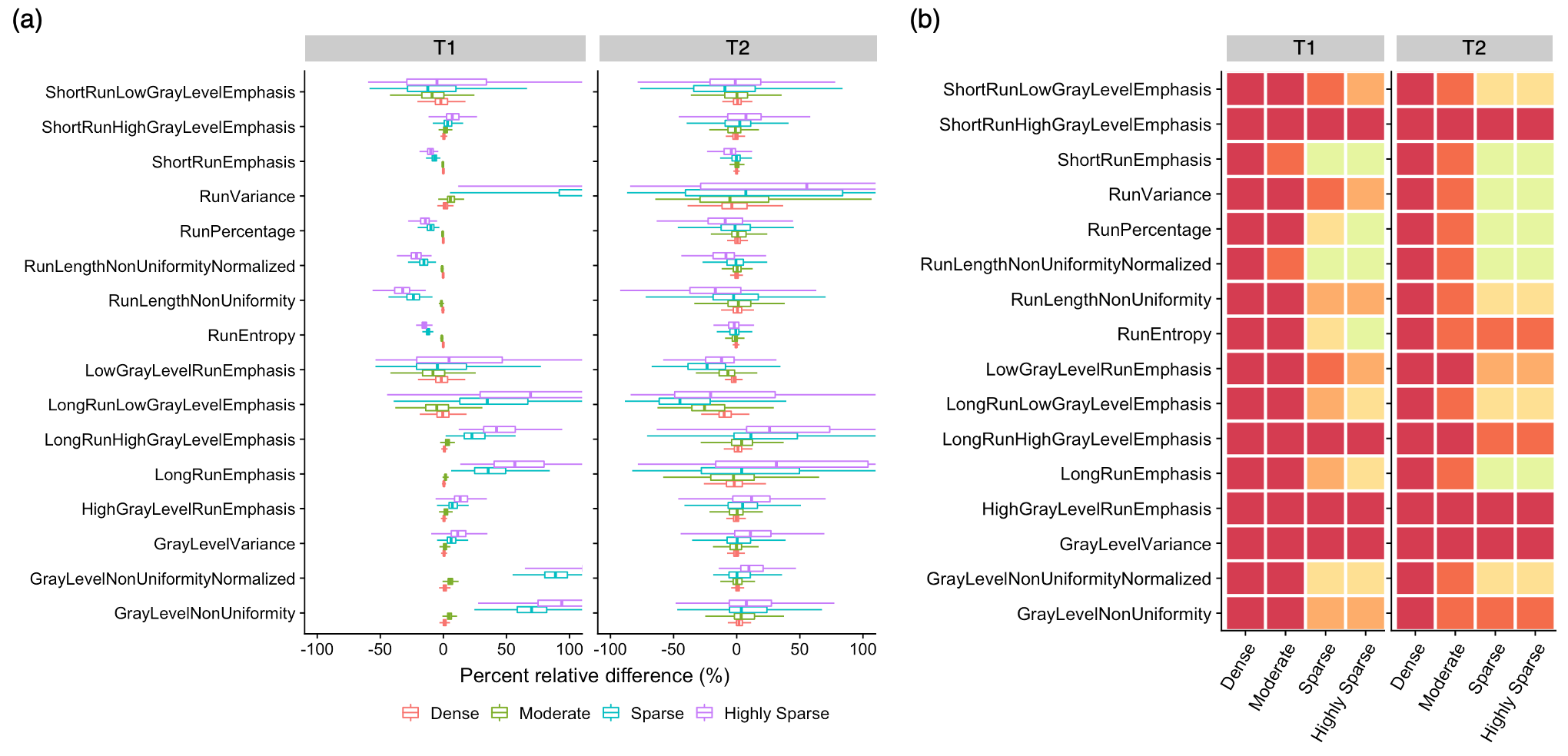


**Electronic Supplementary Material Figure 5.** Effect of magnetic resonance fingerprinting dictionary step size on gray level size zone matrix. (a) Inter-dictionary percent relative changes of features. (b) Intraclass correlation coefficients (ICCs) for each feature (rows) extracted using different dictionary step sizes (columns).


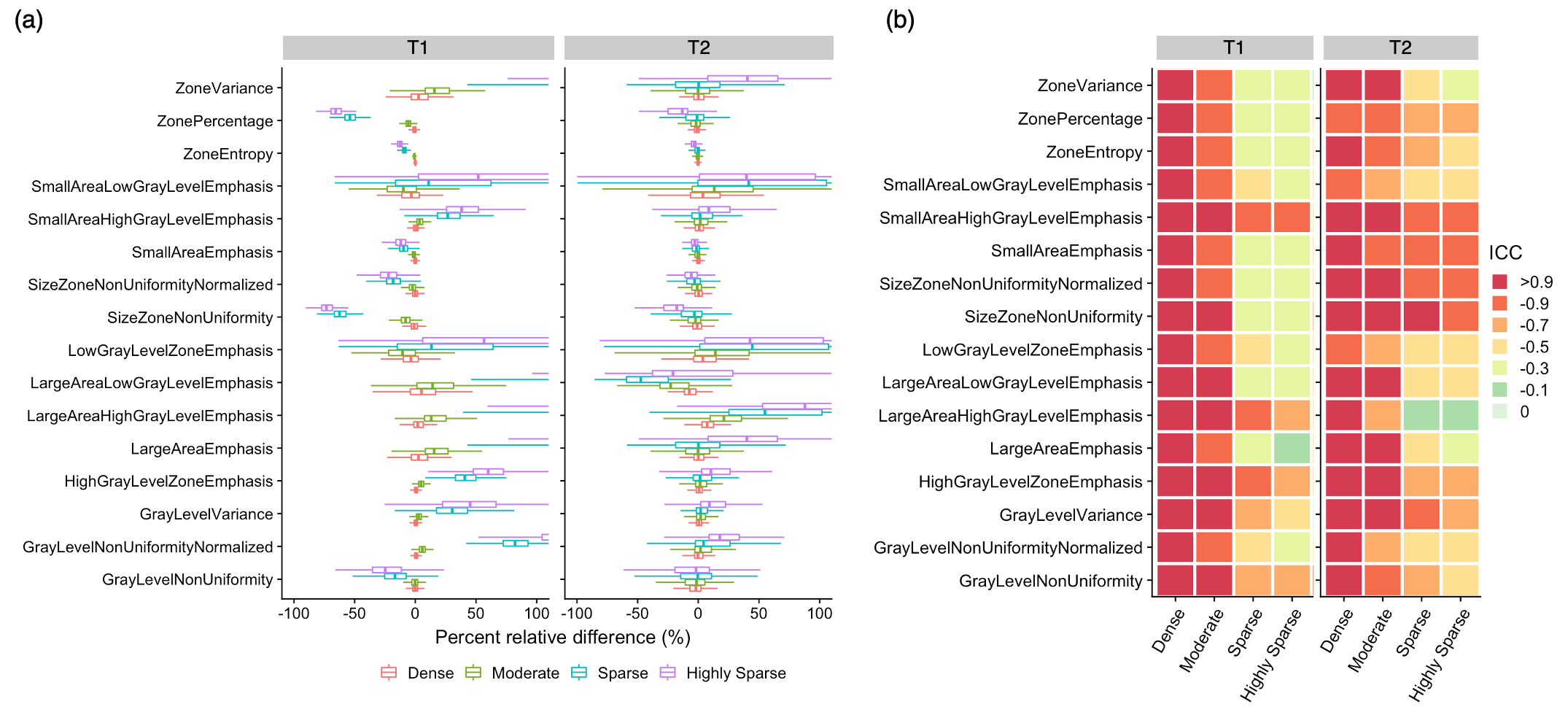


**Electronic Supplementary Material Figure 6.** Effect of magnetic resonance fingerprinting dictionary step size on neighboring gray tone difference matrix. (a) Inter-dictionary percent relative changes of features. (b) Intraclass correlation coefficients (ICCs) for each feature (rows) extracted using different dictionary step sizes (columns).


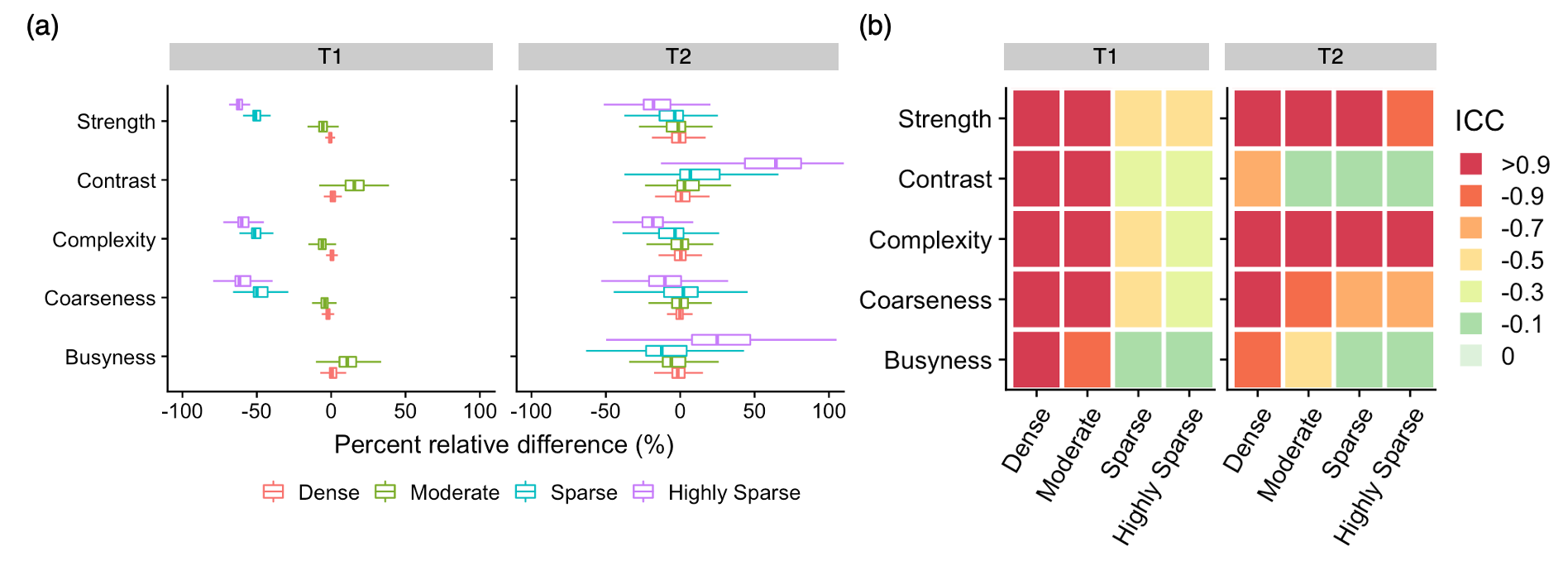


**Electronic Supplementary Material Figure 7.** Inter-dictionary reproducibility of radiomic features calculated with magnetic resonance fingerprinting (MRF) in patients with multiple sclerosis. (a) Representative T1 map and volume of interest placement. (b) Inter-dictionary reproducibility of radiomic features was evaluated with intraclass correlation coefficients computed for the entire study population using features obtained from the highly dense dictionary as a reference. Boxes indicate the interquartile range (25–75%), and circles indicate radiomic features.
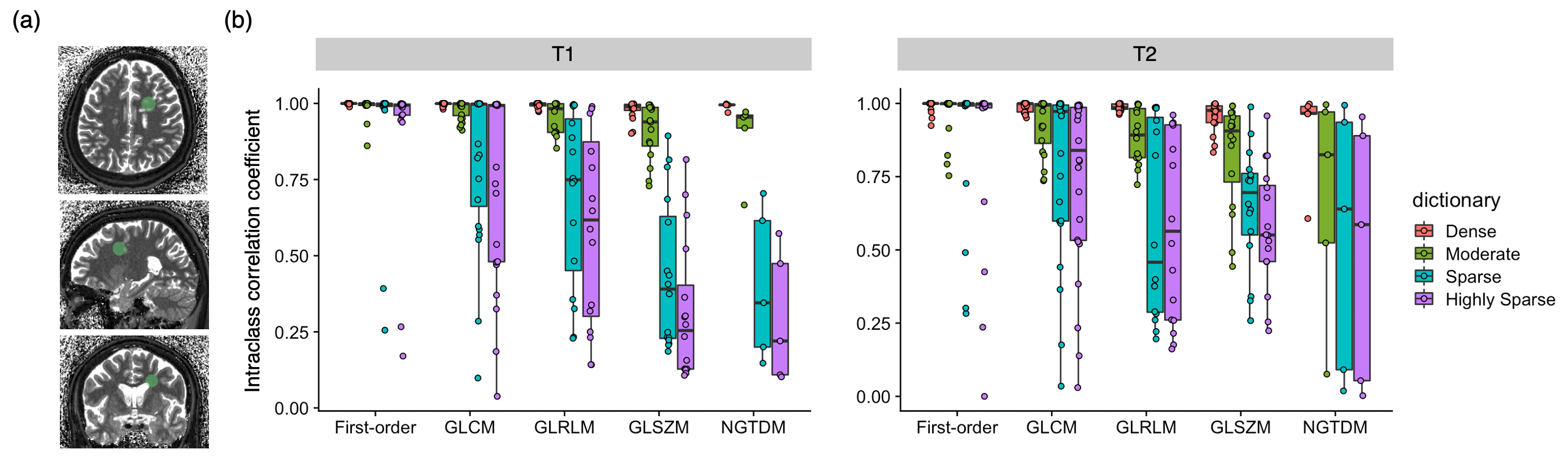

Supplement: Supplementary file 1 — (DOCX 3768 kb) [file 330_2022_8555_MOESM1_ESM.docx]
